# Dissipation signals due to lateral tip oscillations in FM-AFM

**DOI:** 10.3762/bjnano.5.213

**Published:** 2014-11-10

**Authors:** Michael Klocke, Dietrich E Wolf

**Affiliations:** 1 Department of Physics, University of Duisburg-Essen and CeNIDE, D-47048 Duisburg, Germany

**Keywords:** atomic force microscopy (AFM), frequency-modulated atomic force microscopy (FM-AFM), energy dissipation

## Abstract

We study the coupling of lateral and normal tip oscillations and its effect on the imaging process of frequency-modulated dynamic atomic force microscopy. The coupling is induced by the interaction between tip and surface. Energy is transferred from the normal to the lateral excitation, which can be detected as damping of the cantilever oscillation. However, energy can be transferred back into the normal oscillation, if not dissipated by the usually uncontrolled mechanical damping of the lateral excitation. For certain cantilevers, this dissipation mechanism can lead to dissipation rates larger than 0.01 eV per period. The mechanism produces an atomic contrast for ionic crystals with two maxima per unit cell in a line scan.

## Introduction

The usage of scanning probe microscopes requires an understanding of the physical processes during the scan, otherwise images can be misinterpreted. Due to the importance of frequency-modulated atomic force microscopy (FM-AFM), the physical processes involved have been studied intensively in the past [1]. This includes the relation between tip–surface interaction and frequency-shift [2], as well as features such as the energy dissipation during the scan [3], which is an interesting side-effect of the FM-AFM principle. The height (the topography) of a point on the surface is measured by shifting the probe such that the resonant frequency of the cantilever oscillation is detuned by a given amount due to surface–tip interactions. The amplitude is kept constant, which requires to drive the oscillation. Energy loss of the oscillation occurs not only due to mechanical damping of the cantilever, but also due to interaction between tip and surface, so that the damping signal can be used for imaging, even with atomic resolution [4].

There is a broad consensus, that the observed dissipation is due to adhesion hysteresis [5]: As the tip approaches the surface, the atomic configuration becomes metastable and at the same time a modified configuration becomes energetically more favorable. When the energy barrier between these two configurations is low enough, a sudden transition into the favorable one happens. The energy difference is dissipated. This mechanism has been found in various systems [6–8].

The ultimate goal would be to extract valuable information about the surface from the rate of the dissipated energy, e.g., the identification of functional groups within molecules [9]. Unfortunately, the effects that take place are far more complex than having just a single hysteresis loop. Depending on the indentation depth of the tip (or the minimum distance between tip and sample), multiple adhesion hysteresis loops might occur [10]. It can also happen that in some of the cycles, there is no adhesion hysteresis loop at all, which leads to complex beating phenomena [11]. Moreover, alternative dissipation effects have been discussed, and it is possible that these effects are additionally at work [12–15]. In this paper, we propose another dissipation mechanism, which can enhance the dissipation signal independently of the presence of adhesion hysteresis.

We show that the bending mode of the cantilever (oscillation normal to the substrate) is coupled to lateral tip oscillations, and connect this coupling to the damping of the cantilever oscillation. The lateral tip oscillations can include torsional or lateral cantilever modes, which also lead to an oscillation of the end of the tip parallel to the surface of the substrate. While torsional cantilever deflections can be detected directly by using techniques similar to the ones used for lateral-force AFM measurements [16–18], this is in general not the case for lateral tip deflections without cantilever torsion. The damping that we are going to describe indicates such deflections.

Up to now, the effect of a lateral displacement of the tip has only been studied for the quasi-static case [19–20]. These studies are valuable for the understanding and correction of certain distortions seen in the actual images. Here, we enhance these studies by taking lateral dynamics of the tip into account and study the effect on the topography as well as on the energy dissipation signal.

It is necessary to distinguish between the terms damping and dissipation. Damping means, that the normal oscillation of the cantilever is reduced. The reason can be irreversible energy dissipation, or a redistribution of energy between normal and lateral modes. In principle, such a redistribution is reversible, but the lateral mode is no perfect energy storage. Mechanical damping is responsible for the dissipation of energy of the macroscopic degrees of freedom. We will therefore address three questions: (i) How large is the damping rate, (ii) is the resulting dissipation rate comparable to adhesion hysteresis, and (iii) what happens to the non-dissipated energy?

The mechanical damping of the normal oscillation mode can be measured directly. It is a common assumption, that the lateral and bending modes of the cantilever are decoupled, but this only holds as long as there are no asymmetries in the mass distribution of the cantilever [21]. We will neglect this kind of coupling, as it is an intrinsic feature of the cantilever and should not be sensitive to the surface potential (we also neglect any kind of direct coupling between different lateral modes [22]). Based on this assumption, we present a simple two-dimensional model for surface-induced coupling between normal and lateral tip oscillations. Later on we use more realistic potential energy landscapes from molecular dynamics calculations. The simulations show, under which circumstances the coupling of normal and lateral modes is strong enough to compete with dissipation rates due to adhesion hysteresis and the effects it has on the imaging process.

## Description of the two-dimensional model

We start from the common one-dimensional description of the cantilever dynamics. The tip is described as a single point of mass *m_z_* [23]. The bending mode oscillation of the cantilever is replaced by the oscillation of a harmonic spring with the spring constant *k_z_*. The mass *m_z_* has to be chosen such that the frequency 
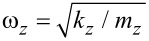
 matches the frequency of the cantilever [24]. The internal damping of the cantilever motion is experimentally compensated by a driving force. There exist sophisticated models that describe the dynamics of an AFM in great detail by taking into account all electrical and mechanical components [25]. The AFM setup itself can also lead to some sort of spurious dissipation signal [26–27]. These effects can additionally be at work, but will not be considered further as they have been sufficiently elaborated [28]. Instead we focus solely on the proposed effect and therefore use a rather simple yet quite often used approach by neglecting both damping and excitation [29]. It is possible to include dissipation caused by the adhesion hysteresis effect by using non-conservative forces [30], but we do not consider this effect here. We define *z* to be the position of the end of the tip. *z*_0_ is the equilibrium position, that is the position when there is no cantilever deflection. Let the interaction between tip and sample be given by a potential energy *V*_ts_. The equation of motion then reads

[1]
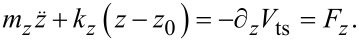


**Figure 1:**
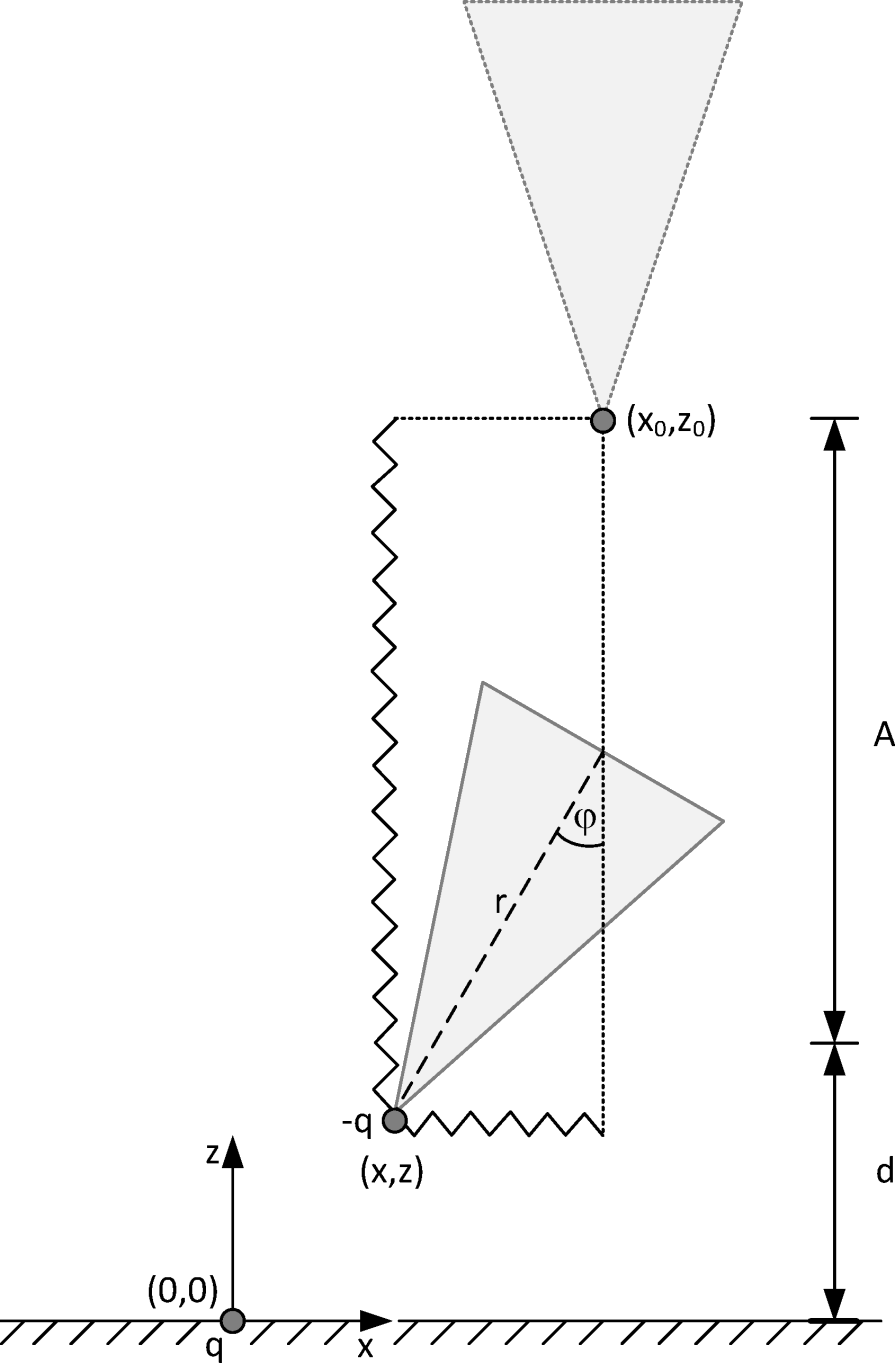
Sketch of the two-dimensional model setup.

This model can easily be extended to include additional degrees of freedom. For simplicity we add only a single lateral displacement coordinate, *x* − *x*_0_, in the plane perpendicular to the cantilever axis, where *x*_0_ denotes the equilibrium value of the *x*-coordinate of the end of the tip in the absence of interaction with the surface, see Figure 1. In analogy to Equation 1 the effective dynamics for the *x*-coordinate is given by

[2]
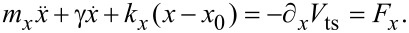


For the lateral excitations we include a viscous friction term with the coefficient *γ*, because these losses are not compensated like they are in the bending mode. Commonly, *γ* is expressed by the quality factor *Q* = (*m_x_ω_x_*)/*γ*, where 
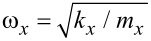
. We only consider damping in the lateral degree of freedom and therefore omit any sort of indexing of damping parameters (such as *Q_x_*). It should be clear that if the damping in the *z*-direction is explicitly used, one will have to distinguish between *Q_x_* and *Q_z_*.

Without distinguishing further degrees of freedom, these equations of motion describe the combined elastic response of cantilever and tip. A recent innovation in FM-AFM is the use of tuning fork cantilevers. Their spring constants are several orders of magnitude larger than those of ordinary cantilevers. In this case, the lateral displacement is mostly due to elastic deformation of the tip. For illustrative purposes we briefly want to discuss the opposite limit, where the lateral displacement originates from a torsion of the cantilever superimposed on its bending. Then *x* − *x*_0_ ≈ *rφ*, where *r* is the length of the tip and *φ* the torsional angle of the cantilever. Denoting the moment of inertia of the cantilever by *J* and the torsional spring constant by *k_φ_*, the equation of motion for *φ* without the interaction with the substrate would be 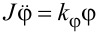, which is in agreement with Equation 2, if one identifies *k_x_* = *k_φ_*/*r*^2^ and *m_x_* = *J*/*r*^2^. The single mass point at (*x*, *z*) appears to have an anisotropic mass, as *m_x_* ≠ *m_z_*.

The interaction between tip and surface can be described by different models [31]. If both the tip and the substrate are ionic crystals, we can imagine a charge *q* at the surface, which has a fixed position taken as the origin of the coordinate system. The mass point representing the cantilever, including the tip, carries a charge of −*q*. The tip–surface interaction then reads


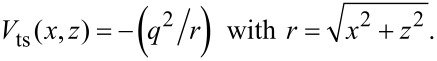


Although this force model is quite simple and might not be as well justified as commonly used models, it provides an important feature that other models lack of: It couples the normal and the lateral motion. For this reason, it seems to be the simplest, yet sufficient, approach to investigate the proposed effect.

The coupled differential equations in Equation 1 and Equation 2 are solved numerically. We use the symplectic velocity-Verlet scheme [32] implemented with our own code; the timestep is set to Δ*t* = 5·10^−5^ (using reduced units in length, frequency and stiffness with *l*_0_ = 10 nm, *ω*_0_ = 100 kHz and *k*_0_ = 0.5 N/m). At the beginning, we place the tip at the upper turning point of the normal oscillation and at the rest position of the lateral spring (so that initially there is no energy in the lateral degree of freedom) with velocity set to zero. We therefore define the amplitude *A* with this initial positioning. The set point *z*_0_ can be used for variations of the distance between tip and sample. We will describe variations of *z*_0_ via the nominal distance *d*, that is the closest distance between tip and sample, if no interaction would take place (*d* = *z*_0_ − *A*, see Figure 1).

Without the damping term (*γ* = 0) in Equation 2, the total energy of the system is conserved. But the total energy is also conserved, if *γ* → ∞, as in this case an excitation of the lateral degree of freedom becomes impossible. For finite *γ*, energy can be transferred into the lateral degree of freedom, but this energy could partly also be transferred back into the normal degree of freedom. The dissipated energy per normal cycle is just given by the viscous damping term in the lateral degree of freedom,

[3]
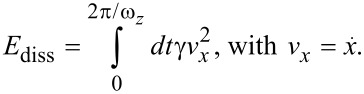


We can estimate the dissipated energy from the energy stored in the lateral spring,

[4]
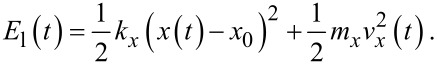


As long as there is no interaction, *E*_l_(*t*) is constant. We denote this constant as *E*_lat_. Assuming that the damping is low and that *ω_x_* >> *ω_z_*, we can integrate Equation 3 for one cycle in *z* with *v_x_* = *v*_0_ cos(*ω_x_t*) and 
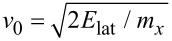
, which yields

[5]
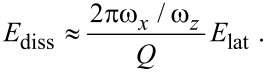


If we just consider one oscillation cycle in our numerical simulations, we start at the upper turning point of the normal oscillation but the lateral excitations set in at the lower turning point. Thus the dissipation values will be just half of the values expected from Equation 5. If *Q* is sufficiently high, some significant amount of energy remains in the lateral degree of freedom after one cycle. This would create a new situation, because it is not clear, how much energy is transferred to the lateral degree of freedom in the succeeding cycles.

**Figure 2:**
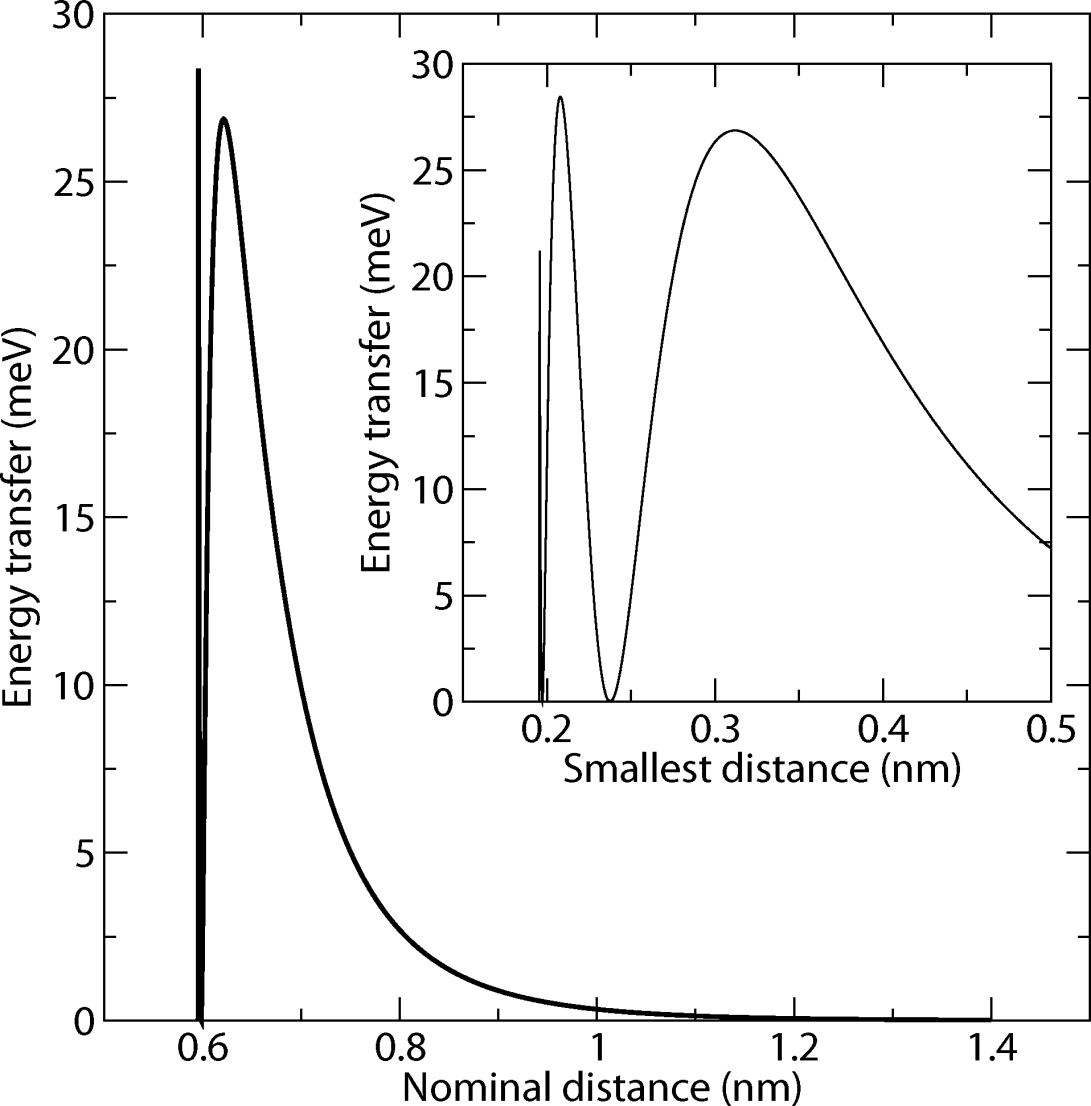
Distance-dependency of the energy transfer after one cycle. As a function of the nominal distance (which would be the closest approach of the tip, if the interaction with the sample was switched off, cf. Figure 1), only one of the oscillations can be resolved. Due to the attraction between tip and sample the tip actually comes closer than the nominal distance. In the inset, which shows the same data, advantage has been taken of the non-linear dependence of the actual minimal distance on the nominal distance in order to resolve a second oscillation.

In the next two sections (and in Figure 2–Figure 4 and Figure 6) we use the following parameters: *k_x_* = 24 N/m, *k_z_* = 0.5 N/m, *ω_x_*/2*π* = 600 kHz, *ω_z_*/2*π* = 100 kHz, *Q* = 30000. The lateral distance between the tip and the fixed charge is set to *x*_0_ = 0.3 nm, the amplitude *A* is set to 3.155 nm. We do not give a default value for the nominal distance here. The values for *k_x_* and *ω_x_* are typical for torsional cantilever oscillations. The actual lateral motion is not necessarily torsional, but can for instance be a local tip deformation. Likewise, *k_z_* and *ω_z_* vary significantly for different cantilevers [33]. In fact, for FM-AFM usually much stiffer cantilevers are used (*k_z_* ≈ 40 N/m) [34] in order to suppress thermal fluctuations and to increase the robustness with respect to snap into contact. This is not so urgent for computer simulations, where the parameters can be explicitly chosen such that the tip keeps oscillating (see e.g. [35]). Then, however, in order to compare with experiments, it is important to show robustness of the simulation results with respect to variations of *k_z_*. The sensitivity with respect to all parameters (including the lateral quality factor *Q*) will be assessed in the later sections.

## Results and Discussion

### Dissipation spectroscopy

#### Energy transfer within one cycle

As a first application of the model, we calculate the energy transfer from the normal into the lateral degree of freedom for different nominal distances. The motion starts at the upper turning point of the normal oscillation. When the cantilever returns to this turning point next time, a part, *E*_lat_, of its energy has been diverted from the bending mode into the lateral degree of freedom, and some energy *E*_diss_ has been dissipated.

Figure 2 shows *E*_lat_ as a function of the nominal distance. Although the interaction with the substrate monotonously increases the closer the tip gets to the surface, the energy *E*_lat_ transferred into the lateral degree of freedom shows an oscillatory behavior.

In order to understand the origin of this oscillating behavior, we will simplify the equations of motion, such that they become analytically solvable. As a first step, the *x*-dependence of the *z*-component of the force is neglected by approximating *F_z_*(*x*, *z*) ≈ *F_z_*(*x*_0_, *z*). Then the solution *z*(*t*) of Equation 1 is independent of Equation 2. This leads to an effectively time-dependent lateral force *F_x_*(*x*, *z*(*t*)) for Equation 2.

As the interaction between the surface and the tip is short ranged, the lateral force may be assumed to be non-zero only for the small fraction *p* of the normal cycle, when the tip is sufficiently close to the surface. We use the following approximation:

[6]
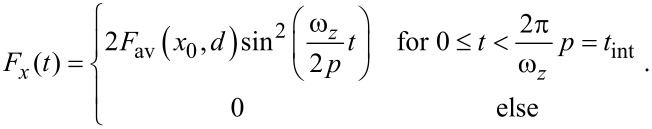


*F*_av_(*x*_0_, *d*) is the lateral force averaged over the time interval *t*_int_, during which it is non-zero. It increases, the smaller the closest nominal approach *d* to the surface becomes.

With this approach, the equation of motion

[7]
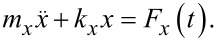


can be solved analytically. We introduce the dimensionless lateral elongation ξ = x/*λ* by using the length scale *λ* = *F*_av_/*k_x_*, the dimensionless time *τ* = *ω_x_t*, and abbreviate the dimensionless duration of interaction by 2*πα* = *ω_x_t*_int_. In these variables, the equation of motion simply reads


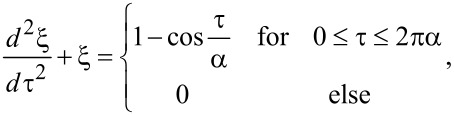


which is solved by


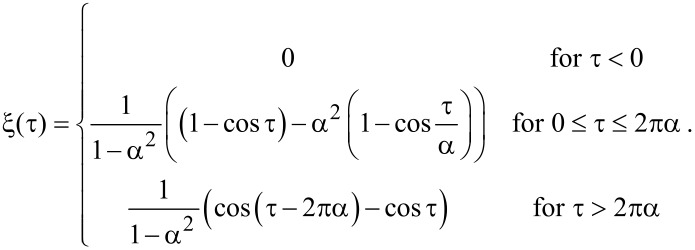


The energy transferred into the lateral degree of freedom after one cycle is

[8]
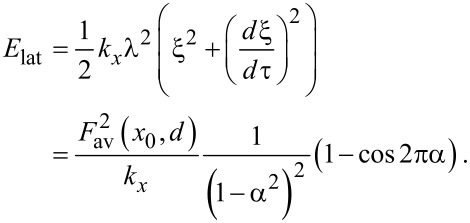


In spite of its simplicity, this model can explain a number of qualitative findings for the numerical solution of the coupled Equation 1 and Equation 2.

When the nominal distance *d* is decreased, the average force *F*_av_ as well as the fraction of time *p* with significant lateral interaction, and hence *α*, increase. While the first leads to a higher amount of transferred energy, varying *α* leads to oscillations as observed in the numerical solution, Figure 2. For *α* = *n* (with any natural number *n*), the lateral excitation is zero, because the energy transiently transferred from the normal into the lateral mode is reversibly given back, when the tip retreats. Note that due to *p* < 1 the number of these oscillations is limited by *α*_max_ = *ω_x_*/*ω_z_*.

**Figure 3:**
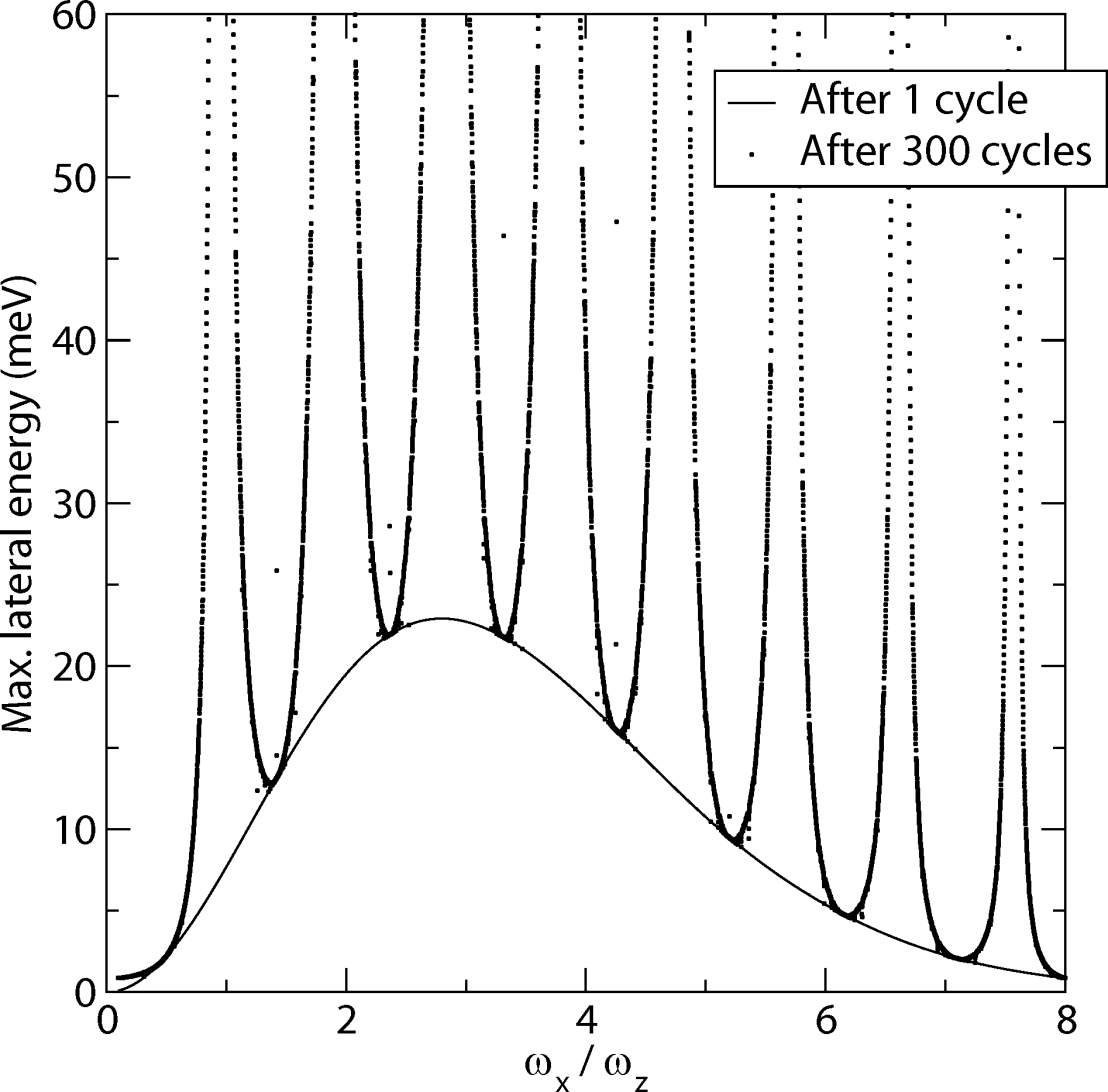
Numerical results of energy in lateral degree of freedom after one cycle (line) and maximum energy in the lateral degree of freedom (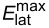) over 300 cycles (points) versus frequency ratio *ω_x_*/*ω_z_*.

The dependence of the energy transfer in a single cycle on the frequency ratio *ω_x_*/*ω_z_* is shown for the numerical solution as the full curve in Figure 3. Like the simplified result in Equation 8 it starts at 0 for *ω_x_*/*ω_z_* = 0, then goes through local maxima decreasing in height with increasing *ω_x_*/*ω_z_* (only first maximum shown). Note that the singularity of (1 – *α*^2^)^−2^ in Equation 8 is removed by the zero of the second factor at *α* = 1. The transferred energy vanishes asymptotically for *ω_x_*/*ω_z_* → ∞ as expected, because the lateral displacement becomes zero for infinite lateral stiffness.

#### Energy transfer and dissipation after multiple cycles

After we have discussed the energy transfer into the lateral degree of freedom within one cycle, we can now consider the actual dissipation rate after multiple cycles. We evaluate the dissipation rate by the loss of total energy of the system divided by the number of normal cycles. Compared to the energy transfer per cycle *E*_lat_ of the previous section, we find values, which are about three orders of magnitude lower, in agreement with Equation 5.

**Figure 4:**
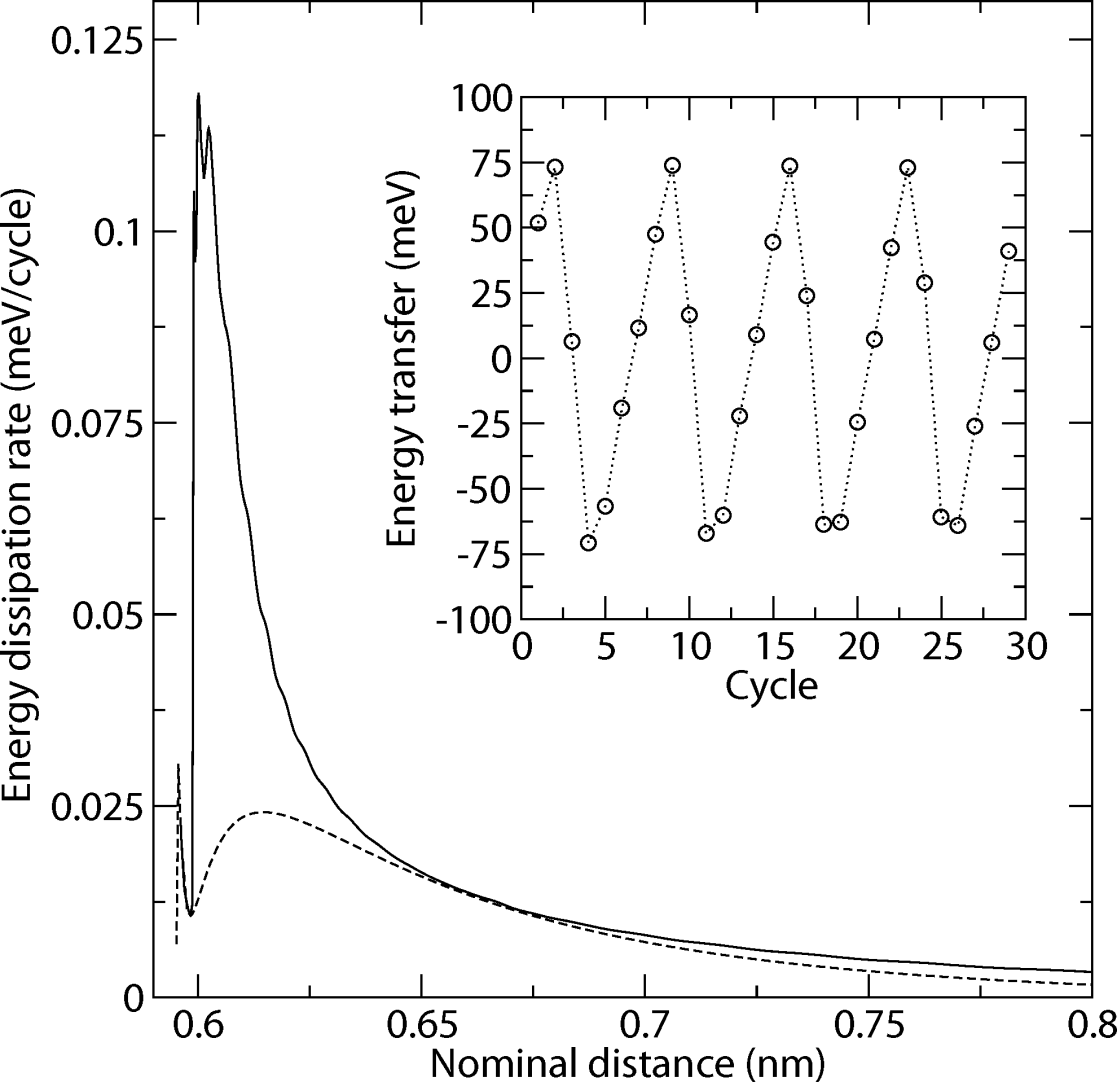
Distance dependency of dissipation rate for one cycle (dashed line) and averaged over 30 cycles (solid line). Inset shows the energy transfer per cycle for the nominal distance *d* = 0.605 nm.

We compare the dissipation rate for one and for 30 cycles. For certain nominal distances, there is only a small observable difference, while for others the dissipation rate for 30 cycles is about five times higher (Figure 4). We also observe the non-monotonic *d*-dependence corresponding to the one derived for *E*_lat_ in the previous section. The lateral excitation can be amplified in succeeding cycles, if the normal oscillation is in resonance with it. We therefore evaluated the energy transfer during each cycle (by evaluating the difference between the lateral energies *E*_l_ in the upper turning point of the normal oscillation for two successive cycles). We found, that the energy transfer oscillates (see inset of Figure 4). As long as it is positive, the lateral oscillation is amplified, but for negative values, energy is transferred back to the normal degree of freedom.

In the previous section, we found with a simple model, that the energy transfer is essentially given by the phase of the lateral oscillation, when the tip leaves the tip–surface interaction region. If we extend the model to a second cycle, we see that the amplification also depends on the phase when the tip enters the interaction region. In Figure 4, interaction times are such that there is a significant energy transfer per cycle, as well as an amplification in the first cycles. After about three cycles the energy transfer changes sign. What may be called an energy swapping frequency, is therefore about *ω_z_*/6 in this case. By altering the nominal distance, we also alter the interaction times. There are points, where there is no difference between the dissipation rate for one cycle and for multiple cycles. At these points, the whole energy stored in the lateral degree of freedom is transferred back in the next cycle (the energy swapping frequency is then *ω_z_*/2).

### Parameter choice

The experimentally observed dissipation rate due to tip–surface interactions in FM-AFM is at least 0.01 eV per cycle. We will now consider the question, what fraction of the observed dissipation rate may be accounted for by the excitation of lateral tip oscillations.

#### Monte-Carlo study of parameter set

Before we study the influence of single parameters, we want to get a quick overview, what can actually be expected. This is obtained by a Monte Carlo sampling of the parameter space, in which we chose combinations of parameters randomly within a reasonable range.

The normal spring constant *k_z_* of typical cantilevers ranges from 0.004 N/m to 40 N/m (for tuning forks it may be 100 times larger). Values for lateral stiffness *k_x_* ranging from *k_z_* up to 1000*k_z_* should cover most cases. For the lateral frequency *ω_x_* we consider a range from *ω_z_* to 30*ω_z_*. The Q-value ranges from 100 to 30000. We also vary the values of *x*_0_ and *z*_0_ between 0 and 0.5 nm and between 10 and 12 nm, respectively, in order to have different ratios between lateral and normal force. The amplitude is always equal 10 nm. We discard parameter sets for which the reduced frequency shift [2] lies outside the interval −7 fN m^1/2^ and −0.7 fN m^1/2^. For each parameter set, we calculate 300 cycles.

**Figure 5:**
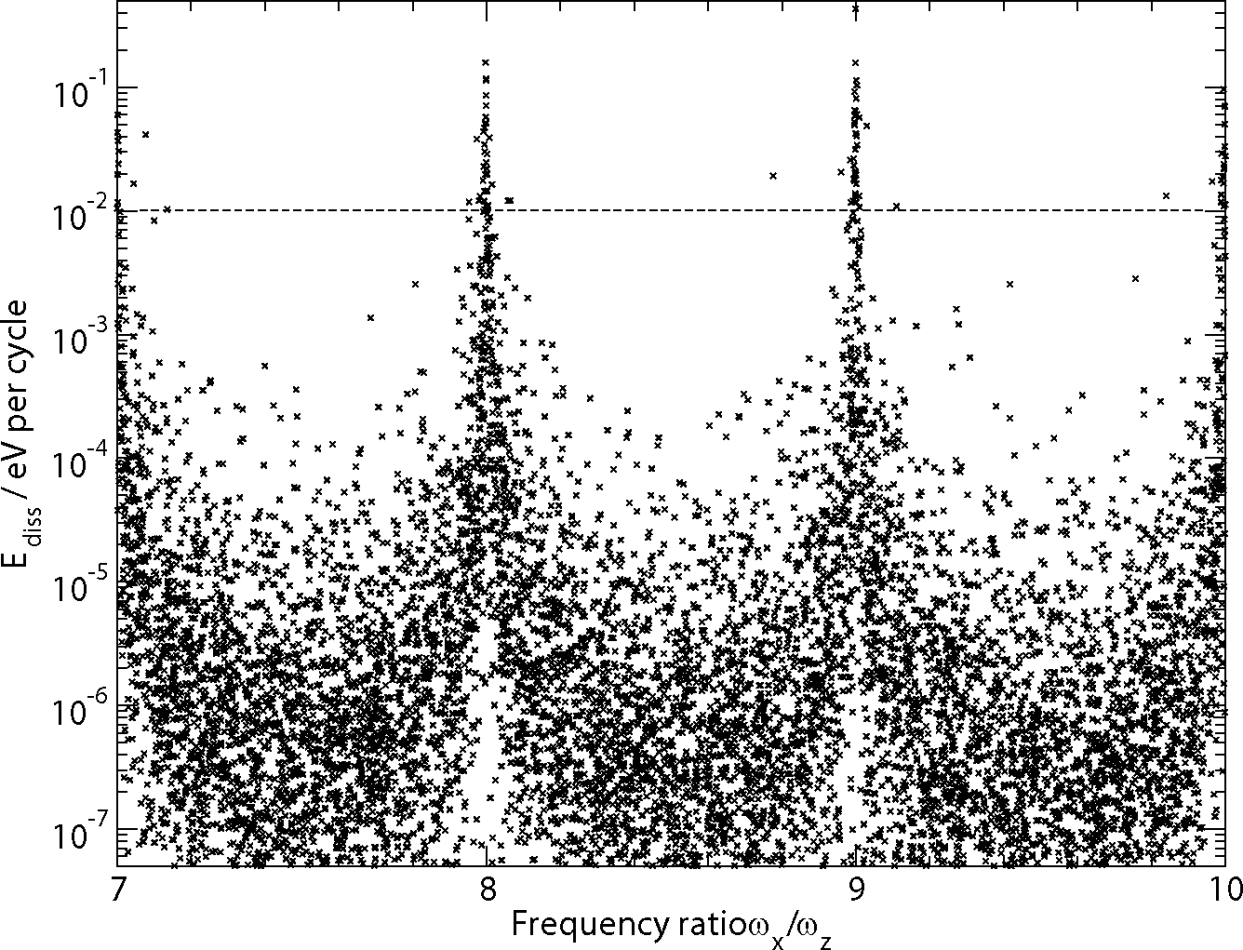
Dissipation rate depending on frequency ratio *ω_x_*/*ω_z_* for different systems. Dashed line indicates the lower bound of experimentally observed values. Dissipation rate is averaged over 300 cycles.

Figure 5 shows that for most parameter sets the energy dissipation due to coupling between normal and lateral oscillations is below 0.01 eV per cycle, but for (nearly) integer ratios of *ω_x_*/*ω_z_* the dissipation rate becomes rather large due to a resonant excitation of the lateral degree of freedom.

#### Lateral frequency

The Monte-Carlo study shows, that the dissipation rate depends strongly on the lateral frequency. To study this effect in detail, we use the parameter set given at the end of the Section in which the model is described. The nominal distance is set to *d* = 0.79 nm, while we vary the lateral frequency *ω_x_*. We perform one simulation with one normal cycle and one with 300 normal cycles.

We evaluate *E*_lat_ after each cycle (in the upper turning point of a normal oscillation) and take the maximum over all 300 normal cycles, denoted by 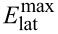. We found that after the first cycle *E*_lat_ has a maximum for a frequency ratio of *ω_x_*/*ω_z_* ≈ 3 (Figure 3), which is quite near the frequency ratio of the torsional and normal frequency for many cantilevers [36]. This value can serve as a lower bound for 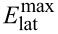 after multiple cycles. There we found resonance-like peaks at nearly integer values of *ω_x_*/*ω_z_*. This indicates that although the dissipation rate after one cycle may be small, higher dissipation rates can be achieved due to further amplification of the lateral oscillation (assuming a relation between 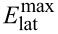 and *E*_diss_ such as in Equation 5). We also observe deviations from a smooth curve, especially at the minima. These are based on a subtle effect, which is caused by the variation of the potential between two consecutive cycles due to the loss of energy in the normal degree of freedom. We will, however, not discuss this effect in detail, as its impact is limited to very narrow ranges of frequency ratios.

#### Quality factor

**Figure 6:**
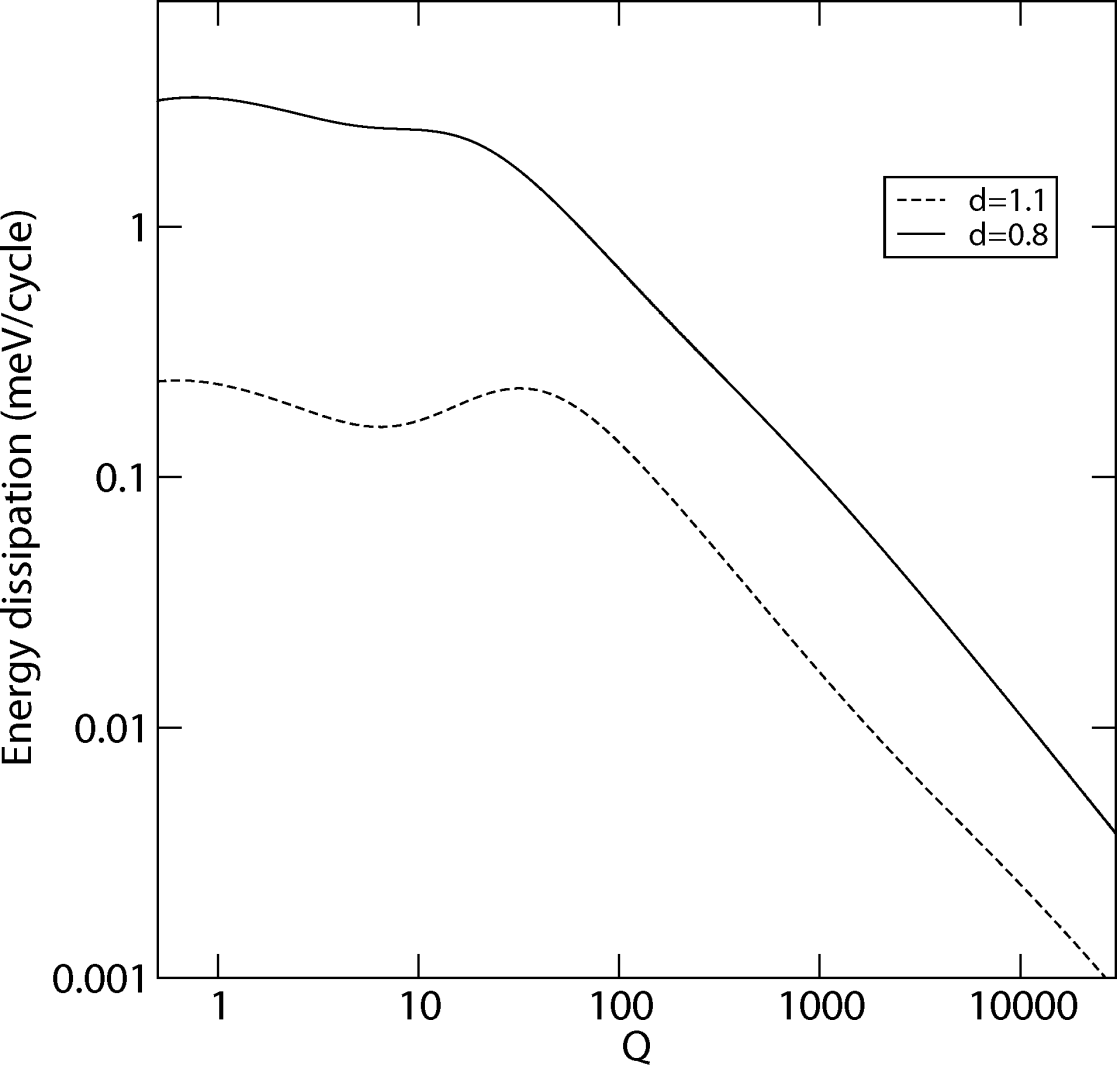
Dissipation rate with respect to the variation of the Q-factor for nominal distances of *d* = 0.8 nm and *d* = 1.1 nm. Numerical simulation for 300 normal cycles with the default parameter set.

We studied the influence of the quality factor on the energy dissipation rate (Figure 6). For high *Q*-values, the trajectory of the tip in the strong interaction region is nearly unaffected by the damping in the lateral degree of freedom. Therefore, the approximation leading to Equation 5 is applicable, which explains the *Q*^−1^-dependence shown in Figure 6. At lower *Q*-values, the effect on the trajectory becomes stronger. The energy transfer into the lateral degree of freedom slows down and, being limited in time by the normal cantilever oscillation, does not reach the level any more that it had for large *Q*. *E*_lat_ decreases with decreasing *Q* so that *E*_diss_ no longer increases like *Q*^−1^. For weak lateral interaction (*d* = 1.1) *E*_diss_ even drops with decreasing *Q*. Heading towards the overdamped case (*Q* < 1), the dissipation rate rises again, as a higher fraction of the transferred energy is actually dissipated. For *Q* = 0 one would not expect any dissipation, as any lateral displacement is suppressed. Figure 6 shows the numerical results for two different nominal distances and the parameter set given at the end of the Section in which the model is described. While the absolute maximum is found for rather low *Q*-values (*Q* ≈ 0.6), a second maximum is found for values of *Q* of about 10–30. Typical quality factors for the normal cantilever oscillation range from 200 [17] up to 100000 [37]. However, the *Q*-values for the lateral oscillations may be smaller.

### Realistic potentials

In the previous sections we have used two point charges as a simple model for the tip–surface interaction, which includes coupling between a lateral and a normal component. In order to show that this simple model captures relevant physics, in this section we compare it with the results of a more realistic model, where tip and substrate consist of many atoms. The interaction is given by the summation over pair potentials as they are used in molecular dynamics.

In classical molecular dynamics, atomic bonding is described by empirical potentials. We used the potential of Fumi and Tosi [38] with parameters from [39] and van der Waals parameters from [40] for the ionic bonding of KBr. The tip is a cube of 3 × 3 × 3 unit cells (216 atoms) of a NaCl-lattice, while there are 7 × 7 × 3 unit cells (1176 atoms) for the substrate. Compared to the crystallographic orientation of the substrate, the tip cube is rotated by the Euler angles *π*/2, 0, and 
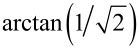
, so that its diagonal axis is parallel to the z-axis. In this configuration there is exactly one apex atom closest to the substrate.

In this setup, the dissipation mechanisms of adhesion hysteresis [6] or more rare transitions between a number of tip configurations [14,41] are known to occur. Here, we want to study the possibility of an additional dissipation mechanism caused by the excitation of lateral oscillations. Therefore, we calculate the tip–substrate interaction force only for the approach of the tip, and assume the same force for the retreat, thereby intentionally suppressing the adhesion hysteresis effect as well as configurational changes. In a first step, we put all atoms on ideal NaCl-lattice positions and run a relaxation separately for the tip and the substrate. Then the two subsystems are assembled with fixed atomic positions of the lowest atomic layer of the substrate and the uppermost atom of the tip cube. The other atomic positions are relaxed by using open boundary conditions and the conjugate gradient method. The interaction force between tip and substrate is evaluated for a fine grid of tip positions by repeating the relaxation procedure each time. The forces are interpolated linearly for the use in Equation 1 and Equation 2.

In order to simulate an FM-AFM, we start at a certain position (*x*_0_, *z*_0_) and integrate Equation 1 and Equation 2 for 10 cycles. The usual reduced frequency shift is adjusted to a given value *f*_set_,

[9]
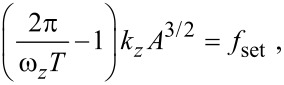


by changing *z*_0_ accordingly. Here, *T* is the actual period of the normal tip oscillation, which due to the attraction by the substrate is larger than 2*π*/*ω_z_*. Afterwards, we displace the tip by small amounts Δ*x*_0_ and determine the topography signal *z*_0_(*x*_0_) for the same reduced frequency shift. The parameter set for the simulation is given in Table 1.

**Table 1:** Parameter set for simulations with KBr potential, and ranges for MC parameter sampling.

quantity	AFM simulation	ranges for MC

*k_x_*	10 N/m	0.05–5000 N/m
*k_z_*	0.5 N/m	0.5–500 N/m
*ω_x_*/2*π*	455 kHz	10–1000 kHz
*ω_z_*/2*π*	100 kHz	100 kHz
*A*	3.0 nm	0.5–6.0 nm
*Q*	50	10–900
*z*_0_	7.10 nm	4–11 nm
*f*_set_	−6.2 fN m^1/2^	
normal cycles	10	200

**Figure 7:**
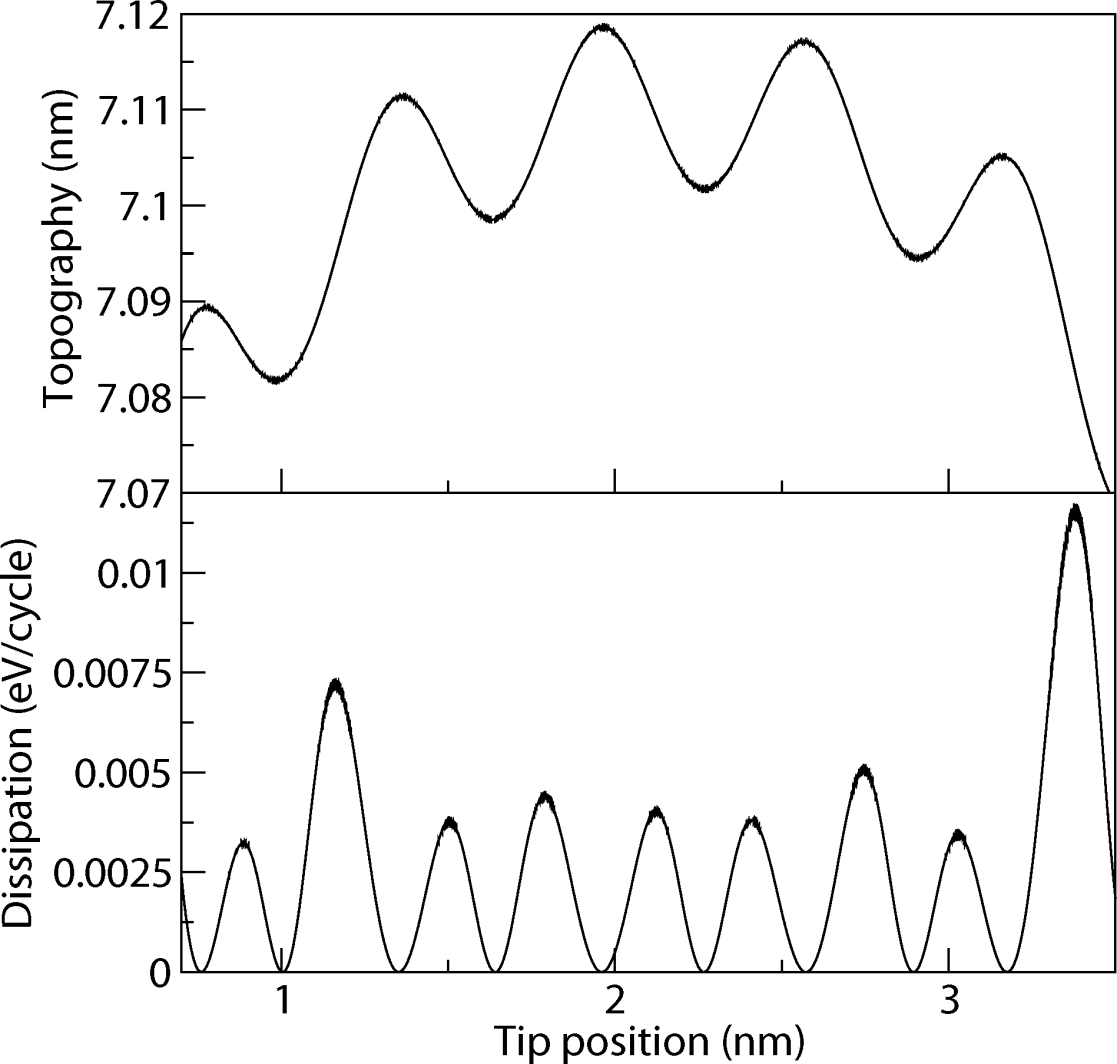
Topography and dissipation for KBr. Scan line along [010]. We observe a doubling in the dissipation signal.

The atomic contrast in the topography signal can be seen clearly (Figure 7). As we used open boundary conditions for the relaxation of the substrate atoms, we have a small distortion at the boundary of the substrate. The surface is therefore not flat, but convex, which is also visible in the topography signal. Such finite size effects are expected to occur not only in the simulation but for real nanostructured surfaces as well.

We see a strong contrast in the dissipation signal. The dissipation signal is slightly below the experimentally observed 0.01 eV per cycle. However, it should be noted, that the frequency ratio is chosen such that a moderate resonance is responsible for the rather high dissipation rate (which means that with a slightly detuned frequency, one would exceed the threshold of 0.01 eV per cycle, but one could also get lower values). It shows two maxima to the left and to the right of one maximum in the topography (comparable to the experimental finding in [42]). As the lateral force is responsible for the dissipation here, these maxima occur where the partial derivative of the potential energy in lateral direction is highest. Due to the boundary-induced distortion of the surface, the maxima at the border of the substrate appear much larger, because in addition to the gradient between the atoms, there is also a displacement in *z*-direction which leads to higher lateral forces.

By choosing a very stiff lateral spring with a high frequency, we can suppress the excitation of lateral oscillations. While the dissipation signal vanishes, the topography is almost unchanged in the present case, because the lateral displacement was smaller than 0.1 nm anyway.

In order to check whether there are higher dissipation rates possible, we performed a Monte-Carlo parameter study again, but now with the potential for KBr. We place the tip such that we expect a maximum in the dissipation signal. We vary the parameter set according to Table 1.

**Figure 8:**
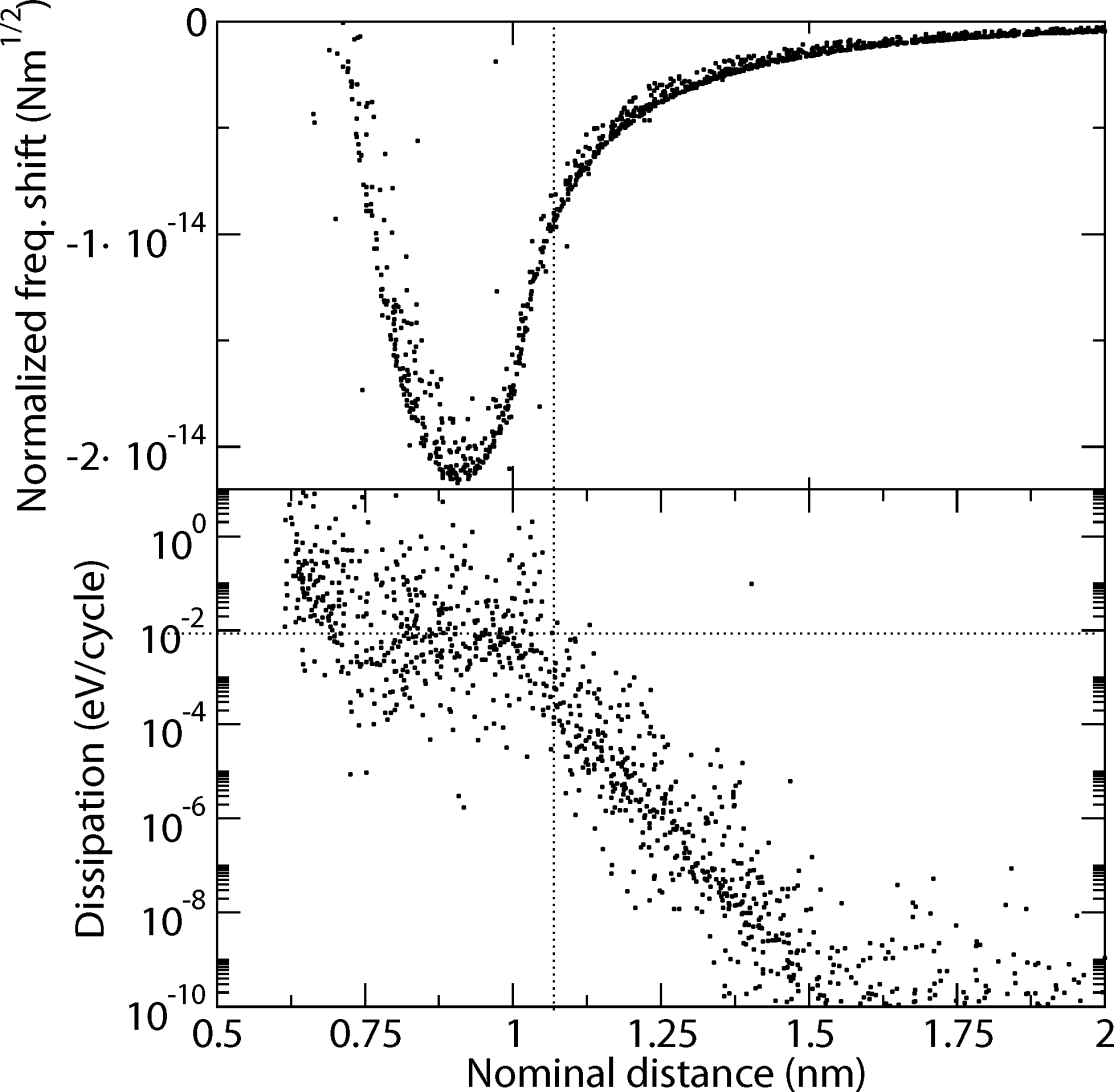
Topography and dissipation for Monte-Carlo parameter-space study.

Figure 8 shows that the dissipation rate increases dramatically by a factor of 10^8^, when the nominal distance decreases from 2 nm to 1 nm. The vertical line roughly indicates a threshold for the nominal distance, such that for distances below that threshold it is likely to find dissipation rates above 0.01 eV per cycle. This threshold corresponds to a certain value for the nominal frequency shift of around 8 fN m^1/2^, which is not uncommon for experimental setups.

## Conclusion

The dissipation signal is an interesting property of the scanning process, but its interpretation is not trivial. In addition to the adhesion hysteresis mechanism, lateral excitations can be responsible for dissipation rates of the same order of magnitude. The strength of the dissipation depends on the absolute value of the lateral forces. One, therefore, expects high dissipation rates at step edges. If atomic resolution is achieved, this dissipation mechanism would show two maxima accompanying one maximum in the topography signal.

The higher the nominal frequency shift is, the closer the tip gets to the surface and lateral forces increase. The Monte-Carlo study showed, that not all conditions have to be met in order to find dissipation rates higher than 0.01 eV per cycle. There is another condition acting here: The relative phase shift of the lateral oscillation between two successive normal cycles plays an important role. The relative phase shift strongly depends on the frequency and the duration, where lateral forces are significantly acting on the tip. The lateral excitation can be amplified over many successive normal cycles thus leading to much higher dissipation rates.

From Equation 8 it can be concluded that the higher the stiffness of the cantilever is, the smaller the energy dissipation gets. It is therefore less probable for cantilevers with high stiffness, such as tuning forks, that the effect shows up. This is a possible reason, why lateral tip displacements had no effect on the dissipation signal in [43].

Although the origin of this dissipation mechanism is based on lateral forces near the surface, the signal cannot be used to quantitatively determine these forces. Even if all parameters of the lateral oscillator would be known, due to the dependency of the dissipation signal on the phase shift, there is no monotonic dependency of the dissipation signal on the lateral force.
